# Law Enforcement Officers’ Ability to Recognize Emotions: The Role of Personality Traits and Basic Needs’ Satisfaction

**DOI:** 10.3390/bs12100351

**Published:** 2022-09-22

**Authors:** Aiste Dirzyte, Faustas Antanaitis, Aleksandras Patapas

**Affiliations:** 1 Institute of Psychology, Mykolas Romeris University, Ateities 20, LT-08303 Vilnius, Lithuania; 2 Institute of Public Administration, Mykolas Romeris University, Ateities 20, LT-08303 Vilnius, Lithuania

**Keywords:** emotion recognition, basic psychological needs, personality traits, law enforcement officers

## Abstract

Background: This study intended to explore the role of personality traits and basic psychological needs in law enforcement officers’ ability to recognize emotions: anger, joy, sadness, fear, surprise, disgust, and neutral. It was significant to analyze law enforcement officers’ emotion recognition and the contributing factors, as this field has been under-researched despite increased excessive force use by officers in many countries. Methods: This study applied the Big Five–2 (BFI-2), the Basic Psychological Needs Satisfaction and Frustration Scale (BPNSFS), and the Karolinska Directed Emotional Faces set of stimuli (KDEF). The data was gathered using an online questionnaire provided directly to law enforcement agencies. A total of 154 law enforcement officers participated in the study, 50.65% were females, and 49.35% were males. The mean age was 41.2 (age range = 22–61). In order to analyze the data, SEM and multiple linear regression methods were used. Results: This study analyzed variables of motion recognition, personality traits, and needs satisfaction and confirmed that law enforcement officers’ personality traits play a significant role in emotion recognition. Respondents’ agreeableness significantly predicted increased overall emotion recognition; conscientiousness predicted increased anger recognition; joy recognition was significantly predicted by extraversion, neuroticism, and agreeableness. This study also confirmed that law enforcement officers’ basic psychological needs satisfaction/frustration play a significant role in emotion recognition. Respondents’ relatedness satisfaction significantly predicted increased overall emotion recognition, fear recognition, joy recognition, and sadness recognition. Relatedness frustration significantly predicted decreased anger recognition, surprise recognition, and neutral face recognition. Furthermore, this study confirmed links between law enforcement officers’ personality traits, satisfaction/frustration of basic psychological needs, and emotion recognition, χ2 = 57.924; df = 41; *p* = 0.042; TLI = 0.929; CFI = 0.956; RMSEA = 0.042 [0.009–0.065]. Discussion: The findings suggested that agreeableness, conscientiousness, extraversion, and neuroticism play an essential role in satisfaction and frustration of relatedness needs, which, subsequently, link to emotion recognition. Due to the relatively small sample size, the issues of validity/reliability of some instruments, and other limitations, the results of this study should preferably be regarded with concern.

## 1. Introduction

Many studies analyzed the behavior of law enforcement officers who have the right to use force to prevent law violations and protect the interests of the individual and society [1]. However, the factors contributing to the officers’ decision on whether the use of force is appropriate in a particular situation are still under-researched [2].

Officers’ use of excessive force can negatively affect public attitudes towards law enforcement institutions [3,4]. When a law enforcement officer’s actions cause violence, it can give rise to mistrust in the authorities [5].

Using excessive force by officers can have highly negative consequences, so it is essential to find out how to reduce it. Previous research explored possible contributing factors such as personality traits, organizational culture, the relationship between officers and civilians [6], the subculture of law enforcement officers [7], cultural context [8], racism [9], and training of officers [10], among others.

Recent studies have demonstrated that violent behavior might be related to emotion recognition [11,12,13,14]. However, the findings are contradictory, and there is still no consensus on why some officers use excessive force and others do not.

This study aims to assess the relationship between the ability to recognize emotions, personality traits, and the satisfaction of basic psychological needs in a sample of law enforcement officers. The research question was whether personality traits and the satisfaction of basic psychological needs are associated with the ability of law enforcement officers to recognize emotions.

### 1.1. Emotions and the Ability to Recognize Emotions

Research on basic emotions has mainly focused on facial expressions, arousal of specific neural networks, emotion-specific autonomic responses, unique subjective experiences, and specific responses to motivational and regulatory functions [15,16,17,18]. Research revealed that each of the six basic emotions (anger, joy, sadness, fear, surprise, disgust) represents a group of related emotions [19], and emotion recognition is related to different brain parts, mainly the amygdala, the anterior cingulate cortex, and the anterior part of the frontal lobe, among others [16,20,21].

Research demonstrated that an emotionally significant stimulus would receive more attention, even with limited cognitive resources [22]. Faces showing positive or negative valence emotions are noticed more rapidly than those with neutral expressions, and stimuli that can cause fear are noticed more quickly [23].

Many studies have confirmed that emotions can affect behavior [24]. When a person feels anger, one may behave more aggressively than usual, focus on anger-related information, be more likely to be guided by prejudices, and make more risky decisions [25,26].

Furthermore, fear and anxiety can affect human decision-making [27]. People who feel sad tend to be more cautious [25] and pessimistic [28]. Fearful people tend to be pessimistic and perceive a situation as threatening, dangerous, unclear, or out of control [28]. Due to fear, the perceived risk increases [29]. Anxiety lowers self-confidence and impairs information processing capabilities [30]. An anxious person is more likely to view an uncertain situation as a potentially dangerous threat [31].

When a person experiences an emotion, the facial expression may indicate the emotion experienced [32,33,34,35]. Although the representation of emotions in the face is widely acknowledged, some authors argue that it is not a universal representation [36] nor an expression of emotionally motivated behavior [37]. Despite criticism, facial expressions allow people to recognize emotions [38].

Recognizing emotions was an evolutionary factor that facilitated survival [39,40]. According to the Theory of Mind, poor emotional recognition could significantly impair social relationships and functioning [41]. Demonstrating positive emotions can help create a positive image and thus encourage collaboration [39]. High levels of emotional recognition can help avoid potential danger and aid survival [42,43].

Some research has highlighted the role of mirror neurons in emotion recognition [40,42,44]. Individuals unconsciously replicate the emotion observed in other people’s faces, but a person may also attribute the wrong emotion to someone’s facial expression [38,45]. A person demonstrating a facial expression associated with a particular emotion can arouse that emotion [46,47].

Emotions are recognized primarily by evaluating valence and arousal dimensions [48]. Each emotion has specific, culture-independent facial features, and changes in facial expression make emotions recognizable [48,49,50]. While people can distinguish one prototypical emotion from another, intermediate expressions are not well-distinguished [51,52], indicating some particular patterns [53].

To sum up, the ability to accurately recognize the emotions of others is critical to successful human survival and interpersonal communication. Recognition of emotions can help prevent situations in which a person may be in danger. Misrecognized emotions can contribute to the escalation of conflict and impair communication. Therefore, exploring factors related to the ability to recognize emotions is very important.

### 1.2. Personality Traits and Ability to Recognize Emotions

Personality is the dynamic organization of physical and psychological systems that determine behavior, thinking, and emotions [54,55]. The five-factor personality model, or the Big Five, validated in clinical, industrial, organizational, consulting, and other contexts [56], lists the five broadest personality traits: extraversion, neuroticism, openness to new experiences, conscientiousness, and agreeableness [57].

Extraversion is a person’s tendency to engage in social relationships to enjoy the attention received [58] and is also characterized by social dominance, emotions of positive valence, and physical activity [59]. Neuroticism means seeing the world as threatening and disturbing, being more anxious, sensitive to stress, easily frustrated, and insecure in relationships [60]. The trait of openness to new experiences is sometimes associated with intelligence [61] and manifests in valuing creativity, diversity, and change [62]. The agreeableness trait is described as having empathy and compassion when asked for help [63] and the ability to suppress emotions of negative valence [54]. The trait of conscientiousness is described as having cautious planning skills, perseverance, a desire to achieve goals [64], keeping order, being responsible, and having reasonable impulse control [65].

Research has indicated that personality traits are relatively stable; although extraversion and neuroticism slightly decrease, agreeableness and conscientiousness increase over time [66,67,68].

Recent studies proposed links between personality traits and personality disorders. Personality disorders are long-term, dysfunctional features of a person’s behavior that strongly influence the person’s functioning in personal, social, and other contexts [69]. The recent classification of personality disorders is based on the five-factor personality model [70]. Research revealed that the features listed in the five-factor personality model are related to different dimensions of personality disorders [71,72,73,74].

Borderline personality disorder manifests as instability in interpersonal relationships, moods, self-image, and impulsivity, which begin in early adulthood and appears in different contexts [75]. This personality disorder is most strongly associated with neuroticism [76]. Research revealed that individuals with this personality disorder poorly recognize emotions in a social context when contextual hints need to be followed [77] or have difficulties recognizing basic emotions, particularly anger, disgust [78], or neutral facial expressions [79].

The narcissistic personality disorder manifests as a person’s grandiosity, the need for admiration, and a lack of empathy that begins in early adulthood [75]. Research revealed that narcissistic personality disorder is associated with personality traits of neuroticism and extraversion [80] and poor recognition of emotions, especially fear and disgust [81].

An antisocial personality disorder is described as ignoring or violating the rights of others, disobeying social norms, and being deceptive, impulsive, and aggressive [75]. This disorder is associated with low agreeableness, conscientiousness, and neuroticism [82]. Individuals with antisocial personality disorder poorly recognize emotions of anger, sadness, and fear [83,84].

Alexithymia is an inability to express, describe, or distinguish emotions and may occur in conjunction with other disorders [85]. People with alexithymia are seen as cold, withdrawn, avoiding social relationships and conflicts, not trying to meet other people’s expectations [86]. Alexithymia is positively associated with neuroticism and negatively associated with openness to new experiences and extraversion [87,88]. Hereditary factors play an essential role in alexithymia [89]. Alexithymic individuals have a poorer ability to distinguish emotions based on facial expressions [90,91].

Despite many attempts to establish the links, the under-researched question is whether personality traits and disorders are linked to the ability to recognize emotions. Matsumoto and colleagues found a positive relationship between the ability to recognize emotions, openness to new experiences, and conscientiousness. However, in the same study, no association was observed between the ability to recognize emotions and extraversion and neuroticism [92]. Jenkins found an association between conscientiousness, extraversion, openness to new experiences, and the ability to recognize emotions [93]. Calder and colleagues suggested a possible link between extraversion and the ability to recognize emotions, but other personality traits were not related to emotion recognition [94]. Terracciano and colleagues demonstrated a link between the ability to recognize emotions and openness to new experiences [95]. Mill and colleagues demonstrated that openness to new experiences positively relates to the ability to recognize emotions [96]. Andric and colleagues revealed a negative association between the trait of neuroticism and the recognition of the emotion of joy [97].

To sum up, the findings of previous studies are inconsistent, and the links between personality traits and emotion recognition still require more detailed research.

### 1.3. Basic Psychological Needs’ Satisfaction and Ability to Recognize Emotions

Research suggests that if the basic psychological needs are satisfied, the situation will be perceived as controlled and pleasant, and if the needs are unmet, the situation will be perceived as uncontrolled and unpleasant [98,99]. When basic psychological needs are unsatisfied, a person may feel anxious, stressed, and more aggressive [100].

The construct of basic psychological needs (competence, relatedness, autonomy) emerged within the self-determination theory [101]. In this theory, the need for competence means a wish to be productive and achieve goals [102]. The need for autonomy is described as a person’s determination and willingness to control one’s life [102]. The need for relatedness means a wish to belong and have a close connection with others [103,104].

Satisfaction of basic psychological needs is associated with better mood, vitality, and self-confidence [101,103], which might positively impact functioning and well-being [105]. The frustration of these needs contributes negatively to psychological well-being and can induce the onset of mental disorders [102]. In addition, the frustration of needs can affect compensatory mechanisms [106], distort the perception of anxiety, and affect the ability to recognize emotions [107,108,109,110].

However, the links between need satisfaction and emotional competencies have not been fully confirmed [111]. Research revealed that stress could negatively affect the ability to recognize emotions [112], and the effect of emotions of negative valence is similar [113,114].

Furthermore, some studies revealed links between personality traits and basic psychological needs. Positive associations were found between needs’ satisfaction and extraversion as well as needs’ frustration and neuroticism [115]. Research showed that conscientious people are likely to put more effort into their work and studies, which leads to better outcomes and satisfaction of the need for competence [115]. A person with neurotic traits may find it more challenging to accept other people’s criticisms, contributing to the frustration of the need for autonomy [115].

### 1.4. Links between Law Enforcement Officer’s Ability to Recognize Emotions, Personality Traits, and Basic Psychological Needs

It has already been mentioned that the ability to recognize emotions is essential for both survival and successful interpersonal communication. However, this ability becomes even more critical when we consider the specific context of the law enforcement profession. For example, police officers have a unique role in society—to ensure the security of persons and property and maintain public order. As a result, police officers may find themselves in situations where their safety or lives may be at risk. To protect themselves or others, police officers, seeing that there is no other option, can use force against the person triggering danger [116].

In order to assess whether the use of force in a particular situation is necessary, several factors have to be subjectively assessed: the seriousness of the crime, whether the person is currently posing an immediate threat to the security of the police officer or others, whether the person is resisting arrest [2]. Previous emotional states can affect the assessment of the situation, and the ability to recognize emotions may play a key role.

Research suggests that anger and fear are differently related to threat assessment. Although anger and fear trigger the amygdala, which is associated with a response to a threat, the response to anger and fear is different [117,118]. When someone perceives others’ fear, it can be understood as an indication of some danger and a warning to be careful, so the person will be more attentive, looking for a possible source of threat [118,119]. Observing anger in another person might inform of a sudden, immediate danger [120]. If others’ emotions are misjudged, it may affect the misconduct (e.g., excessive use of force).

Pre-trial investigators can positively use the ability to recognize emotions by conducting interviews and gathering evidence. For example, by successfully recognizing the emotions of others during the interview, they can create a connection that could positively contribute to the quality of the information gathered. Correctional staff could better understand the needs of convicts by recognizing their emotions, thus possibly contributing to the rehabilitation process. These are just a few examples that suggest that the ability to recognize emotions can be beneficial in the work of a law enforcement officer.

Research on law enforcement officers’ personality traits is contradictory. Challacombe and colleagues found no differences in personality traits between law enforcement officers and the general population [121]. However, other studies have found that law enforcement officers score higher on conscientiousness and agreeableness and lower on neuroticism and openness to new experiences [122].

Law enforcement officers’ needs’ satisfaction and frustration are still under-researched. However, some indirect effects might be considered. First, law enforcement officers’ work requires compliance with specific instructions, which may not correspond to officers’ attitudes [123], resulting in frustration of the need for autonomy. Next, officers may find themselves in situations that cannot be resolved (unsuccessful pre-trial investigations, etc.), which can negatively contribute to the satisfaction of the need for competence. Given the peculiarities of officers’ work, it is no surprise that they may find themselves in situations that evoke unpleasant emotions and hamper emotion recognition. Moreover, as mentioned above, negative emotionality can negatively contribute to the quality of interpersonal relationships. As a result, officers may have difficulty maintaining social, non-work-related relationships, making it challenging to meet the need for relatedness.

Based on the studies mentioned in the literature review, we aimed to assess the links between law enforcement officers’ personality traits, satisfaction/frustration of basic psychological needs, and the ability to recognize emotions. Based on the findings of previous studies, we hypothesized that:

**H1**:  *Law enforcement officers’ personality traits play a role in emotion recognition (personality traits are linked to emotion recognition)*;

**H2**:  *Law enforcement officers’ basic psychological needs satisfaction/frustration play a role in emotion recognition (basic psychological needs satisfaction/frustration are linked to emotion recognition)*;

**H3**:  *There are links between law enforcement officers’ personality traits, satisfaction/frustration of basic psychological needs, and emotion recognition*.

## 2. Materials and Methods

### 2.1. Sample

The survey was conducted from 8 March 2022 to 2 April 2022. The survey was conducted in Lithuania. The nationality of the participants was not a factor in the analysis, so it is unknown whether all the participants were of Lithuanian nationality. One hundred fifty-six respondents (law enforcement officers) participated in the study. The data was collected using an online questionnaire. The data of two respondents were removed as those questionnaires were considered corrupt.

The final number of study participants was 154. Of these, 78 (50.65%) were women, and 76 (49.35%) were men. The mean age was 41.2 (age range = 22–61). Six women and ten men had secondary education, fourteen women and eight men had non-university higher education, and 58 women and 58 men had a higher university education. Four women and two men were currently studying; the rest were not. The respondents’ law enforcement activities: (1) police activities, (2) criminal police activities, (3) activities of the State Border Guard Service, (4) activities of the probation service, (5) activities of correctional institutions, (6) activities of pre-trial investigation, (7) activities of the public security service, (8) activities of the prosecutor’s office, (9) activities of the security service, (10) other activities of the law enforcement officer not specified. The minimum work experience was one year, the longest was 32 years, and the average was 16.79 years.

A priori power analysis was performed using G * Power (version 3.1.9.2) to determine the sample size required for this study. Based on studies of the relationship between personality traits and the ability to recognize emotions [92,93,95,96], the required sample size ranged from 27 to 1027 participants.

The study participants were selected by contacting law enforcement institutions directly. The specified emails requesting participation in the ongoing survey were sent. The request provided a brief introduction and explanation of research purposes. The potential benefits of the study were also presented. Finally, given the specific sample required for the study, i.e., law enforcement officers, the survey was not publicly available, meaning that only those who received a study link could participate. Therefore, there is no reason to presume that persons not involved in the activities of law enforcement officials participated in the investigation.

Participation in the study was anonymous and voluntary, and all the respondents provided their consent to participate in the study. The procedure was administered online and followed the General Data Protection Regulation (GDPR) guidelines and the requirements of the Helsinki Declaration. The participants did not receive compensation.

### 2.2. Instruments

This study applied three instruments, the translated Lithuanian version of the Big Five Inventory–2 (BFI-2) [124], the translated Lithuanian version of the Basic Psychological Needs Satisfaction and Frustration Scale (BPNSFS) [102], and the Karolinska Directed Emotional Faces set of stimuli (KDEF) [125]. To ensure that the Lithuanian items correspond as closely as possible to the English items, the original items of instruments were translated into Lithuanian and back-translated.

#### 2.2.1. KDEF

Static and dynamic stimuli may measure different mechanisms underlying emotion recognition ability; however, they both still measure emotion recognition ability. In order to evaluate the emotion recognition ability from facial expressions, static stimuli were applied—colored photographs of emotional faces. The decision to use static stimuli was made based on the work of Calvo and colleagues, who found that mistakes made by the participants in emotion recognition tasks did not significantly differ between static and dynamic stimuli groups. In addition, Khosdelazad and colleagues found that emotion recognition results from facial expression tasks correlate between static and dynamic stimuli groups [126,127].

The Karolinska Directed Emotional Faces set of stimuli was used in the present study. The Karolinska Directed Emotional Faces (KDEF) is a set of 4900 pictures of human facial expressions designed by Lundqvist, Flykt, and Öhman (1998). The set of pictures contains 70 individuals (35 male, 35 female) displaying seven different emotional expressions (angry, fearful, disgusted, sad, happy, surprised, and neutral). Each expression is viewed from 5 different angles [125]. A validation study was conducted by Goeleven and colleagues, who found that the KDEF facial picture database offers a valid set of affective stimuli that can significantly contribute to emotion research [128]. Goeleven and colleagues’ study selected photographs of the best quality based on the correct identification of emotion.

Photographs used in the present study were selected based on Goeleven and colleagues’ findings. In total, 70 photographs were selected. The selection was based on the quality of photographs, meaning that an individual actor may only appear once. Thirty-five photographs were displayed with a male face and thirty-five with a female face. Each facial expression (anger, sadness, disgust, surprise, fear, joy, neutral face) was shown in 10 photographs. This condition could help control the possibility that the emotional faces of some actors are just easier to identify correctly. The time participants could look at the stimuli was not limited, meaning they could look at a photograph for as long as they wanted. Lastly, participants had to choose which emotion was displayed in the photograph from the multiple-choice answers (angry, fearful, disgusted, sad, happy, surprised, and neutral). The choice to use different specific emotions, rather than grouping them into positive and negative valence categories, was made using previous studies, which all had used six basic emotions rather than two categories. The multiple-choice approach was used because it would ease the analysis process.

#### 2.2.2. Big Five-2

In order to evaluate personality traits (extraversion, neuroticism, agreeableness, conscientiousness, and openness to experience), the Big Five Inventory–2 (BFI-2) was applied. This inventory uses 60 items to assess the Big Five personality domains and 15 more specific facet traits [124]. The BFI-2 items are short, descriptive phrases with the shared item: “I am someone who…” (e.g., “Is outgoing, sociable,” “Tends to be quiet”). Respondents rate each item using a 5-point Likert scale ranging from “disagree strongly” to “agree strongly”. The BFI-2 was validated in previous studies [124,129].

#### 2.2.3. BPNSFS

In order to assess law enforcement officers’ needs satisfaction, this study applied the Basic Psychological Need Satisfaction and Frustration Scale (BPNSFS), which assesses satisfaction or frustration of basic psychological needs [102]. This scale of 24 questions assesses satisfaction of needs for autonomy, competence, and relatedness and frustration of these needs arising from their non-satisfaction. This scale has six subscales. The autonomy satisfaction subscale consists of 4 statements, e.g., “I feel that my decisions reflect what I want”. The autonomy frustration subscale consists of 4 statements, e.g., “I feel compelled to do many things I would not want to do”. The relatedness satisfaction subscale consists of 4 statements, e.g., “I feel that the people I care about also care about me”. The relatedness frustration subscale consists of 4 statements, e.g., “I feel separated from the group I want to belong to”. The competence satisfaction subscale consists of 4 statements, e.g., “I feel confident that I can do the job well”. The Cronbach’s alpha for this subscale in this study was 0.85. The competence frustration subscale consists of 4 statements, e.g., “I have serious doubts about whether I can do the job well”. Participants rated each statement on a five-point Likert scale, ranging from 1 (“strongly disagree”) to 5 (“strongly agree “). The BPNSFS was validated in previous studies [102,130].

### 2.3. Data Analysis

For data analysis, we used SPSS v.26.0, JASP v.0.16.1, and Jamovi v.2.2.1. First, we conducted CFA to analyze the validity of the instruments used in this study, then we conducted SEM to analyze the links between the study variables. In the CFA and SEM analyses, model fit was evaluated based on the CFI (Comparative Fit Index), the Tucker–Lewis coefficient (TLI), RMSEA (Root Mean Square Error of Approximation), and SRMR (Standardized Root Mean Square Residual), whereas the χ2 was used for descriptive purposes only [131]. The values higher than 0.90 for CFI and TLI and values lower than 0.08 for RMSEA and SRMR were considered indicative of an acceptable fit [132]. This research considered *p*-values less than 0.05 statistically significant [133]. The data distribution was considered to meet the criteria for normality if skewness and kurtosis estimates did not exceed the range from −1 to 1, despite the Shapiro–Wilk test results [134].

For reliability analysis, Cronbach’s alpha indexes were calculated, and confirmatory factor analysis (CFA) was performed for validity analysis. The Cronbach’s alphas and results of the CFA for the instruments used in this study are presented in Table 1.

The CFA demonstrated an acceptable model fit for some study variables. Unfortunately, some estimates were lower than those reported by the instruments’ authors.

A multiple linear regression (forward method) was also used to test H1 and H2.

## 3. Results

The descriptive statistics on survey variables in the law enforcement officers’ sample are reported in Table 2.

In the preliminary analysis, we have calculated correlations between overall emotion recognition, personality traits, and basic psychological needs’ satisfaction and frustration. The Spearman correlations between the study variables are presented in Table 3.

To test H1, which presumed that law enforcement officers’ personality traits play a role in emotion recognition, we conducted a multiple linear regression using emotion recognition as the criterion and personality traits as predictors (forward method). The results of multiple regression models in the law enforcement officers sample, when the dependent variable is recognition of emotions, and the predictors are personality traits, are displayed in Table 4.

A significant regression equation was found in a group of law enforcement officers regarding overall emotion recognition and agreeableness, F (1.152) =6.060, *p* = 0.015), with an R2 = 0.038. Respondents’ predicted overall emotion recognition was equal to 0.781 + 0.031 (agreeableness) points. Emotion recognition increased by 0.031 points for each agreeableness point. Thus, agreeableness (B = 0.031, *p* < 0.01) contributed significantly to the model and was a significant predictor of increased overall emotion recognition. Furthermore, a significant regression equation was found in a group of law enforcement officers regarding anger recognition and conscientiousness, F (1.152) =5.980, *p* = 0.016), with an R2 = 0.038. Conscientiousness (B = 0.037, *p* < 0.01) contributed significantly to the model and predicted increased anger recognition. Moreover, several significant models were identified regarding joy recognition and personality traits. In model 1, joy recognition was significantly predicted by extraversion, F (1.152) = 4.966, *p* = 0.027), with an R2 = 0.032. In model 2, joy recognition was significantly predicted by extraversion and neuroticism, F (1.152) = 4.669, *p* = 0.011), with an R2 = 0.058. In model 3, joy recognition was significantly predicted by extraversion, neuroticism, and agreeableness, F (1.152) = 4.605, *p* = 0.004), with an R2 = 0.084.

To test H2, which presumed that law enforcement officers’ basic psychological needs satisfaction/frustration play a role in emotion recognition, we conducted a multiple linear regression using emotion recognition as the criterion and satisfaction/frustration of basic psychological needs as predictors (forward method). The results of multiple regression analysis in the law enforcement officers’ sample, when the dependent variable is recognition of emotions and the predictors are satisfaction and frustration of basic psychological needs, are displayed in Table 5.

A significant regression equation was found in a group of law enforcement officers regarding overall emotion recognition, and relatedness needs satisfaction, F (1.152) = 13.868, *p* < 0.001), with an R2 = 0.084. Respondents’ predicted overall emotion recognition was equal to 0.741 + 0.037 (relatedness satisfaction) points. Emotion recognition increased by 0.037 points for each relatedness satisfaction point. Thus, relatedness satisfaction (B = 0.037, *p* < 0.001) contributed significantly to the model and was a significant predictor of increased overall emotion recognition. Next, a significant regression equation was found in a group of law enforcement officers regarding anger recognition and relatedness frustration, F (1.152) = 15.051, *p* < 0.001), with an R2 = 0.090. Respondents’ predicted anger recognition was equal to 1.060–0.043 (relatedness frustration) points. Emotion recognition decreased by 0.043 points for each relatedness frustration point. Relatedness frustration (B = −0.043, *p* < 0.001) contributed significantly to the model and predicted decreased anger recognition. A significant regression equation was found in a group of law enforcement officers regarding sadness recognition, and relatedness needs satisfaction, F (1.152) = 4.677, *p* = 0.032), with an R2 = 0.030. Relatedness satisfaction (B = 0.044, *p* = 0.032) contributed to the model and significantly predicted increased sadness recognition. Next, a significant regression equation was found in a group of law enforcement officers regarding surprise recognition and relatedness frustration, F (1.152) = 5.013, *p* = 0.027), with an R2 = 0.032. Respondents’ predicted surprise recognition decreased by 0.030 points for each relatedness frustration point. Relatedness frustration (B = −0.030, *p* < 0.001) contributed significantly to the model and predicted decreased surprise recognition. Next, a significant regression equation was found in a group of law enforcement officers regarding fear recognition and relatedness satisfaction, F (1.152) = 4.667, *p* = 0.032), with an R2 = 0.030. Relatedness satisfaction (B = 0.083, *p* = 0.032) contributed significantly to the model and predicted increased fear recognition. Then, a significant regression equation was found in a group of law enforcement officers regarding joy recognition, and relatedness needs satisfaction, F (1.152) = 6.535, *p* = 0.012), with an R2 = 0.041. Respondents’ predicted joy recognition was equal to 0.791 + 0.041 (relatedness satisfaction) points. Joy recognition increased by 0.041 points for each relatedness satisfaction point. Thus, relatedness satisfaction (B = 0.041, *p* = 0.012) contributed significantly to the model and was a significant predictor of increased joy recognition. Finally, a significant regression equation was found in a group of law enforcement officers regarding neutral face recognition and relatedness frustration, F (1.152) = 4.259, *p* = 0.041), with an R2 = 0.027. Respondents’ predicted neutral face recognition was equal to 1.014–0.019 (relatedness frustration) points. Neutral face recognition decreased by 0.019 points for each relatedness frustration point. Relatedness frustration (B = −0.019, *p* = 0.041) contributed significantly to the model and predicted decreased neutral emotion recognition.

**Figure 1:**
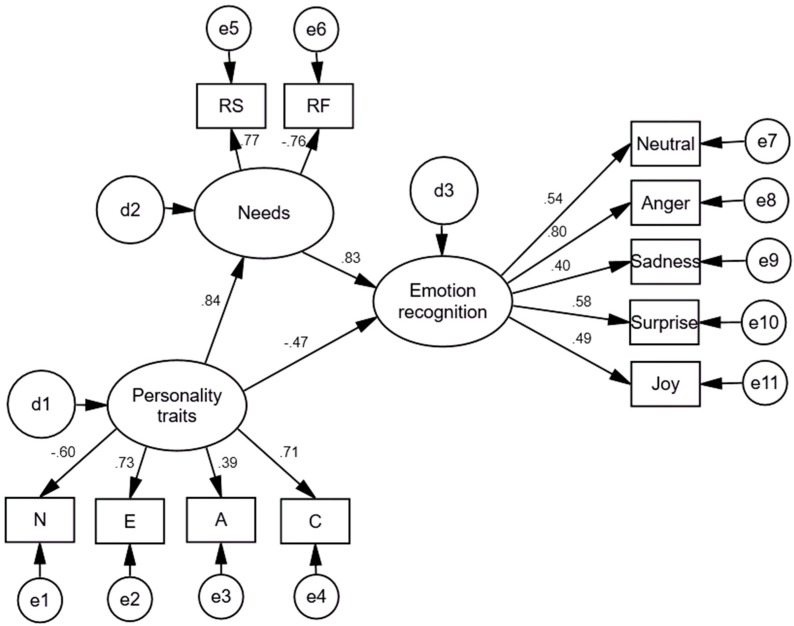
Model on associations between personality traits, (relatedness) need satisfaction and frustration, and emotion recognition in law enforcement officers’ sample. N = neuroticism; E = extraversion; A = agreeableness; C = conscientiousness; RS = relatedness satisfaction; RF = relatedness frustration.

Furthermore, to test H3, which presumed links between law enforcement officers’ personality traits, emotion recognition, and satisfaction/frustration with basic psychological needs, we applied the structural equation modeling (SEM). Based on the results of multiple regression analysis and after testing several alternative SEM models, we created a model on associations between personality traits and relatedness need satisfaction/frustration, and emotion recognition (Figure 1). The model’s fit was good, χ2 = 57.924; df = 41; *p* = 0.042; TLI = 0.929; CFI = 0.956; RMSEA = 0.042 [0.009–0.065].

The scalar estimates of the model on associations between the study variables are presented in Table 6.

The findings suggest that personality traits play an essential role in satisfaction and frustration of relatedness needs, which, subsequently, link to emotion recognition. We must note that we eliminated recognition of disgust and fear recognition in the model due to low factor loadings.

Next, we applied mediation analysis and tested an alternative model based on the literature review and previous analyses. The outcome variable for the mediation analysis was emotion recognition; the predictor was conscientiousness, and the mediator variable was relatedness satisfaction. The mediation analysis results indicating the role of relatedness satisfaction are presented in Table 7. The indirect effects of relatedness satisfaction on emotion recognition were statistically significant (*p* = 0.005). Conscientiousness significantly predicted relatedness satisfaction, predicting emotion recognition, and the total effect was also significant (*p* = 0.047). However, R2 for emotion recognition was just 0.084, and relatedness satisfaction R2 was 0.209.

In summary, this study confirmed that some law enforcement officers’ personality traits and basic needs satisfaction/frustration plays a role in emotion recognition.

## 4. Discussion

This study was possibly the first to explore the role of personality traits and basic psychological needs satisfaction and frustration on emotion recognition, namely, recognition of anger, joy, sadness, fear, surprise, disgust, neutral emotion, and overall emotion recognition. The examination of emotion recognition was based on Ekman and colleagues’ theory of emotional expressions [15]. The analysis of personality traits was based on the Big Five theory and model developed by Soto and colleagues [124]. The analysis of basic psychological needs was based on the self-determination theory, developed by Deci and colleagues [101]. It was significant to analyze law enforcement officers’ emotion recognition and the contributing factors, as this field has been under-researched despite high practical urgency due to increased excessive force use by officers in many countries [6,135,136,137].

### 4.1. Law Enforcement Officers’ Personality Traits Play a Role in Emotion Recognition

In this study, we assumed (**H1**) that law enforcement officers’ personality traits play a role in emotion recognition. Therefore, we conducted a multiple linear regression using emotion recognition as the criterion and personality traits as predictors (forward method). The findings revealed a significant regression equation in a group of law enforcement officers regarding overall emotion recognition and agreeableness. Respondents’ agreeableness was a significant predictor of increased overall emotion recognition. Furthermore, a significant regression equation was found in a group of law enforcement officers regarding anger recognition and conscientiousness. Law enforcement officers’ conscientiousness predicted increased anger recognition.

Additionally, this study identified several significant models regarding joy recognition and personality traits. In model 1, joy recognition was significantly predicted by extraversion. In model 2, joy recognition was significantly predicted by extraversion and neuroticism. In model 3, joy recognition was significantly predicted by extraversion, neuroticism, and agreeableness. These results align with some previous research suggesting that, on the whole, personality traits are linked to emotional experiences and emotion recognition [92]. However, previous studies found no associations between the ability to recognize emotions and extraversion and neuroticism [92] or, on the contrary, demonstrated links between only trait extraversion and the ability to recognize emotions [94], which is just partly supported by our findings.

Furthermore, previous research demonstrated links between neuroticism and recognition of joy [97], which is the opposite of our results. In addition, previous research identified links between conscientiousness, extraversion, openness to new experiences, and the ability to recognize emotions [93], which is partly in line with our results. Next, several studies demonstrated a significant link between the ability to recognize emotions and the personality trait of openness to new experiences [95,96], but in our study, openness to new experiences did not contribute significantly to any model. To sum up, the findings of this study support research implying that certain personality traits predict increased emotion recognition. However, it is still unclear why there were no links between emotion recognition and openness to new experiences, as indicated by other studies, or why some results were just opposite to the previous findings.

### 4.2. Law Enforcement Officers’ Basic Psychological Needs Satisfaction/Frustration Play a Role in Emotion Recognition

Furthermore, we presumed (**H2**) that law enforcement officers’ basic psychological needs satisfaction/frustration play a role in emotion recognition. Thus, we conducted a multiple linear regression analysis in the law enforcement officers’ sample, when the dependent variable was recognition of emotions, and the predictors were satisfaction and frustration of basic psychological needs. The findings revealed a significant regression equation in a group of law enforcement officers regarding overall emotion recognition and relatedness needs satisfaction. Respondents’ relatedness satisfaction significantly predicted increased overall emotion recognition, fear recognition, joy recognition, and sadness recognition. Furthermore, relatedness frustration significantly predicted decreased anger recognition, surprise recognition, and neutral face recognition. These findings complement other studies which non-directly demonstrate the possible importance of basic psychological needs satisfaction and frustration in emotion recognition [39,41,42,43]. However, it is still unclear why in this study, only one basic psychological need (relatedness) contributed to the emotion recognition and the underlying mechanisms of the identified pattern.

### 4.3. There Are Associations between Law Enforcement Officers’ Personality Traits, Satisfaction/Frustration of Basic Psychological Needs, and Emotion Recognition

Based on the literature review and previous analyses, we presumed (**H3**) links between law enforcement officers’ personality traits, emotion recognition, and satisfaction/frustration of basic psychological needs. Based on the results of multiple regression analyses and after testing several alternative SEM models, we created a model on associations between personality traits and relatedness need satisfaction/frustration, and emotion recognition. The model’s fit was good, χ2 = 57.924; df = 41; *p* = 0.042; TLI = 0.929; CFI = 0.956; RMSEA = 0.042 [0.009–0.065]. The findings suggested that personality traits play an essential role in satisfaction and frustration of relatedness needs, which, subsequently, link to emotion recognition. Next, we applied mediation analysis and tested an alternative model based on the literature review. The outcome variable for the mediation analysis was emotion recognition; the predictor was conscientiousness, and the mediator variable was relatedness satisfaction. The findings revealed that conscientiousness significantly predicts relatedness satisfaction, predicting emotion recognition.

On the whole, the findings confirmed that some law enforcement officers’ personality traits (namely, agreeableness, conscientiousness, extraversion, neuroticism) and basic needs satisfaction/frustration (namely, relatedness need) play a role in emotion recognition (namely, overall emotion recognition, recognition of anger, sadness, surprise, joy, neutral emotion) and all the constructs are related. These results partially and non-directly confirm the findings of other authors, evidencing links between needs satisfaction and emotions [92,98,99,113] or associations between personality traits and basic needs satisfaction/frustration [115,138]. However, it is unclear why, e.g., openness to experiences did not contribute to the models, contrary to some findings [93]. Surprisingly, in this study, neuroticism was positively related to joy recognition, which was not in line with other studies [97].

Furthermore, based on previous research, we considered that frustration of basic psychological needs would contribute to a diminished ability to recognize positive emotions and an increased ability to recognize negative ones, as the frustration of basic psychological needs contributes to the emotional state of negative valence, increasing the focus on negative valence emotions. In that case, a positive association between the frustration of those needs and the recognition of negative valence emotions, such as anger or sadness, should be observed [113]. However, this was not confirmed. Next, the satisfaction or frustration of needs for competence and autonomy did not play a significant role in law enforcement officers’ ability to recognize emotions, contrary to what we presumed. Finally, it is somewhat explainable why the need for relatedness was significant and contributed to emotion recognition, but overall the established links between basic needs satisfaction/frustration and emotion recognition raise many questions and require further investigation.

To sum up, the findings of this research can be moderately supported by previous studies indicating the benefits of certain personality traits and psychological needs satisfaction for emotion recognition [99,113]. The added value or risks of personality traits and psychological needs satisfaction/frustration for emotion recognition demands further investigation. In the future, it would be essential to identify the associations between law enforcement officers’ emotion recognition and stress overload, which receives increased attention from researchers.

### 4.4. Limitations and Future Directions

Several limitations to this study could be mentioned. Firstly, the sample size was relatively small (*n* = 154), even though it was sufficient for the applied statistical hypotheses. Preferably, the survey on law enforcement officers’ ability to recognize emotions should be conducted with a larger sample. Second, the findings should be regarded with caution, considering that the data were collected online. In addition, the participants performed the emotion recognition tasks using their computers. Their resolution, screen size, and other technical parameters may vary, so different computer screens may have influenced how well participants perceive stimulus details and, at the same time, emotion recognition. Third, this study was conducted in Lithuania, and the results might reflect the specifics of this area, suggesting the necessity for analyzing the impact of cultural factors, considering the more specific aspects of each society. Fourth, in this survey, the personality traits assessment instrument (Big Five-2) did not demonstrate desired reliability and validity, although it demonstrated good validity and reliability results in Lithuania in different samples [138,139]. It is therefore unclear whether the results of this study correspond to reality or whether they were obtained due to an incorrect instrument measurement. Thus, the results of this study need to be assessed and interpreted in the context of the limitations.

## 5. Conclusions

This study intended to explore the role of personality traits and basic psychological needs in law enforcement officers’ ability to recognize emotions. First, this study confirmed that law enforcement officers’ personality traits play a role in emotion recognition. The findings revealed that respondents’ agreeableness significantly predicted increased overall emotion recognition. Law enforcement officers’ conscientiousness predicted increased anger recognition. Law enforcement officers’ joy recognition was significantly predicted by extraversion, neuroticism, and agreeableness. These findings supplemented the results of previous studies. Second, this study confirmed that law enforcement officers’ basic psychological needs satisfaction/frustration play a role in emotion recognition. Respondents’ relatedness satisfaction significantly predicted increased overall emotion recognition, fear recognition, joy recognition, and sadness recognition. Relatedness frustration significantly predicted decreased anger recognition, surprise recognition, and neutral face recognition. These results also contributed to a better understanding of links between basic psychological needs satisfaction/frustration, and emotion recognition. Third, this study confirmed links between law enforcement officers’ personality traits, satisfaction/frustration of basic psychological needs, and emotion recognition, χ2 = 57.924; df = 41; *p* = 0.042; TLI = 0.929; CFI = 0.956; RMSEA = 0.042 [0.009–0.065]. The findings suggested that agreeableness, conscientiousness, extraversion, and neuroticism play an essential role in satisfaction and frustration of relatedness needs, which, subsequently, link to emotion recognition. These results partially and non-directly confirm the findings of other authors, evidencing associations between needs satisfaction and emotion recognition. However, the added value or risks of personality traits and psychological needs satisfaction/frustration for emotion recognition requires further investigation, preferably including the stress overload factor. Due to the relatively small sample size, the issues of validity/reliability of some instruments, and other limitations, the results of this study should preferably be regarded with concern.

## Figures and Tables

**Table 1:** Cronbach’s alphas and results of the CFA for the Big Five-2 and the BPNSFS.

	χ^2^	df	CFI	TLI	SRMR	RMSEA	Cronbach α
Big Five-2	3309	1700	0.546	0.528	0.116	0.0784	
Extraversion	96.6	51	0.902	0.873	0.057	0.0762	0.8
Agreeableness	180	51	0.683	0.59	0.0949	0.128	0.78
Conscientiousness	90	51	0.909	0.883	0.0588	0.0705	0.82
Neuroticism	128	51	0.862	0.822	0.0992	0.078	0.85
Openness to experiences	96.8	51	0.886	0.852	0.0732	0.0764	0.53
BNSFS	391	237	0.901	0.885	0.0612	0.065	
Autonomy Satisfaction	2.79	2	0.995	0.985	0.0193	0.0506	0.78
Autonomy Frustration	4.11	2	0.982	0.945	0.0293	0.0827	0.72
Relatedness Satisfaction	3	2	0.994	0.982	0.0206	0.0569	0.77
Relatedness Frustration	3.34	2	0.991	0.973	0.0215	0.066	0.76
Competence Satisfaction	9.18	2	0.97	0.91	0.0274	0.153	0.83
Competence Frustration	4.56	2	0.981	0.942	0.0268	0.0912	0.74

**Table 2:** Descriptive statistics and data distribution in the law enforcement officers’ sample.

	Mean	Standard Deviation	Skewness	Kurtosis	Shapiro–Wilk *p*
Age	41.25	9.30	−0.27	−0.72	0.003
Emotion Recognition	62.56	5.19	−2.20	10.57	<0.001
Recognition of Anger	9.77	0.90	−7.08	62.30	<0.001
Recognition of Sadness	8.62	1.48	−1.27	1.39	<0.001
Recognition of Disgust	8.75	1.45	−1.39	1.98	<0.001
Recognition of Surprise	9.33	1.04	−2.00	5.02	<0.001
Recognition of Fear	6.73	2.75	−0.81	−0.11	<0.001
Recognition of Joy	9.59	1.16	−3.24	10.04	<0.001
Recognition of Neutral face	9.76	0.74	−4.16	19.55	<0.001
Extraversion	3.51	0.50	0.02	−0.43	0.55
Agreeableness	3.65	0.47	−0.02	−0.02	0.79
Conscientiousness	3.75	0.47	−0.25	0.33	0.14
Neuroticism	2.63	0.56	0.10	0.00	0.57
Openness to experiences	3.42	0.39	0.17	0.08	0.34
Autonomy Satisfaction	3.83	0.62	−0.07	−0.47	0.01
Autonomy Frustration	2.79	0.73	0.02	−0.03	0.07
Relatedness Satisfaction	4.08	0.57	−0.35	−0.34	<0.001
Relatedness Frustration	1.94	0.63	0.48	0.01	<0.001
Competence Satisfaction	4.15	0.54	−0.11	−0.02	<0.001
Competence Frustration	1.99	0.65	0.58	0.66	<0.001

**Table 3:** The Spearman correlations between the study variables in the law enforcement officers’ sample.

		Emotion Recognition	1	2	3	4	5	6	7	8	9	10
1.	Extraversion	0.041	-									
2.	Agreeableness	0.119	0.256 **	-								
3.	Conscientiousness	0.080	0.562 **	0.341 **	-							
4.	Neuroticism	0.027	−0.419 **	−0.308 **	−0.383 **	-						
5.	Openness to experiences	0.048	0.463 **	0.146	0.275 **	−0.060	-					
6.	Autonomy Satisfaction	0.059	0.605 **	0.249 **	0.385 **	−0.448 **	0.341 **	-				
7.	Autonomy Frustration	−0.079	−0.291 **	−0.370 **	−0.210 **	0.468 **	0.010	−0.435 **	-			
8.	Relatedness Satisfaction	0.177 *	0.523 **	0.276 **	0.451 **	−0.384 **	0.240 **	0.534 **	−0.208 **	-		
9.	Relatedness Frustration	−0.183 *	−0.407 **	−0.301 **	−0.424 **	0.536 **	−0.076	−0.342 **	0.408 **	−0.606 **	-	
10.	Competence Satisfaction	0.022	0.706 **	0.192 *	0.576 **	−0.478 **	0.403 **	0.653 **	−0.253 **	0.607 **	−0.433 **	-
11.	Competence Frustration	−0.052	−0.538 **	−0.296 **	−0.580 **	0.542 **	−0.145	−0.551 **	0.366 **	−0.431 **	0.558 **	−0.667 **

** *p* < 0.001, * *p* < 0.05.

**Table 4:** The multiple regression models in the law enforcement officers’ sample; the dependent variable is recognition of emotions, and the predictors are personality traits.

Dependent Variable	Predictors/ Models	Unstandardized Coefficients	Standardized Coefficients		*t*	Sig.	R	R2	Adjusted R2	F	Sig.
		B	Std. Error	Beta							
Emotion Recognition	Constant	0.781	0.046		16.986	0.000	0.196	0.038	0.032	6.060 (1.152)	0.015
	Agreeableness	0.031	0.013	0.196	2.462	0.015					
Anger Recognition	Constant	0.837	0.058		14.505	0.000	0.195	0.038	0.032	5.980 (1.152)	0.016
	Conscientiousness	0.037	0.015	0.195	2.445	0.016					
Joy Recognition	1 Constant	0.812	0.067		12.210	0.000	0.178	0.032	0.025	4.966 (1.152)	0.027
	Extraversion	0.042	0.019	0.178	2.228	0.027					
	2 Constant	0.654	0.101		6.459	0.000	0.241	0.058	0.046	4.669 (2.151)	0.011
	Extraversion	0.059	0.020	0.252	2.903	0.004					
	Neuroticism	0.037	0.018	0.179	2.065	0.041					
	3 Constant	0.495	0.126		3.929	0.000	0.290	0.084	0.066	4.605 (3.150)	0.004
	Extraversion	0.055	0.020	0.232	2.687	0.008					
	Neuroticism	0.046	0.018	0.219	2.494	0.014					
	Agreeableness	0.042	0.020	0.170	2.068	0.040					

**Table 5:** The multiple regression models in the law enforcement officers’ sample: the dependent variable is recognition of emotions, and the predictors are basic psychological needs satisfaction/frustration.

Dependent Variable	Predictors/Models	Unstandardized Coeff.	Standardized Coefficients		*t*	Sig.	R	R2	Adjusted R2	F	Sig.
		B	Std. Error	Beta							
Emotion Recognition	Constant	0.741	0.041		17.947	0.000	0.289	0.084	0.078	13.868 (1.152)	0.000
	Relatedness Satisfaction	0.037	0.010	0.289	3.724	0.000					
Anger Recognition	Constant	1.060	0.023		47.089	0.000	0.300	0.090	0.084	15.051 (1.152)	0.000
	Relatedness Frustration	−0.043	0.011	−0.300	−3.880	0.000					
Sadness Recognition	Constant	0.680	0.085		8.029	0.000	0.173	0.030	0.023	4.677 (1.152)	0.032
	Relatedness Satisfaction	0.044	0.021	0.173	2.163	0.032					
Surprise Recognition	Constant	0.991	0.027		36.705	0.000	0.179	0.032	0.026	5.013 (1.152)	0.027
	Relatedness Frustration	−0.030	0.013	−0.179	−2.239	0.027					
Fear Recognition	Constant	0.336	0.158		2.128	0.035	0.173	0.030	0.023	4.667 (1.152)	0.032
	Relatedness Satisfaction	0.083	0.038	0.173	2.160	0.032					
Joy Recognition	Constant	0.791	0.066		11.934	0.000	0.203	0.041	0.035	6.535 (1.152)	0.012
	Relatedness Satisfaction	0.041	0.016	0.203	2.556	0.012					
Neutral Recognition	Constant	1.014	0.019		52.661	0.000	0.165	0.027	0.021	4.259 (1.152)	0.041
	Relatedness Frustration	−0.019	0.009	−0.165	−2.064	0.041					

**Table 6:** The scalar estimates of the model on associations between latent factors of personality traits, (relatedness) need satisfaction/frustration, and emotion recognition in the law enforcement officers’ sample.

Variables			Unstandardized Estimates	S.E.	Standardized Estimates	*p*
Personality_traits	→	Needs	1.123	0.167	0.841	0.000
Needs	→	Emotion recognition	0.075	0.033	0.833	0.025
Personality_traits	→	Emotion_recognition	−0.056	0.042	-0.471	0.177
Needs	→	Relatedness frustration	−1.073	0.133	-0.758	0.000
Personality traits	→	Conscientiousness	1.000		0.713	
Personality_traits	→	Agreeableness	0.557	0.130	0.393	0.000
Personality traits	→	Extraversion	1.090	0.148	0.733	0.000
Personality_traits	→	Neuroticism	−1.007	0.160	-0.599	0.000
Emotion recognition	→	Neutral face recognition	1.000		0.538	
Emotion recognition	→	Anger recognition	1.799	0.329	0.800	0.000
Emotion_recognition	→	Sadness recognition	1.498	0.388	0.404	0.000
Emotion recognition	→	Surprise recognition	1.511	0.305	0.578	0.000
Emotion_recognition	→	Joy recognition	1.435	0.322	0.492	0.000
Needs	→	Relatedness satisfaction	1.000	0.046	0.774	

**Table 7:** Mediation analysis results in the sample of law enforcement officers: the role of relatedness satisfaction.

Paths			Coeff.	Std. Error	z-Value	*p*	95% CI Lower Upper	
Direct effects								
Conscientiousness →	Emotion recognition		0.005	0.014	0.373	0.709	0.022	0.032
Indirect effects								
Conscientiousness →	Relatedness satisfaction →	Emotion recognition	0.020	0.007	2.835	0.005	0.006	0.034
Total effects								
Conscientiousness →	Emotion recognition		0.025	0.013	1.984	0.047	0.001	0.050
Emotion recognition R2 = 0.084; Relatedness satisfaction R2 = 0.209.								

## Data Availability

The data presented in this study are available on request from the corresponding author.
